# The Association between Accumulation of Toxic Advanced Glycation End-Products and Cytotoxic Effect in MC3T3-E1 Cells

**DOI:** 10.3390/nu14050990

**Published:** 2022-02-26

**Authors:** Akiko Sakasai-Sakai, Takanobu Takata, Masayoshi Takeuchi

**Affiliations:** Department of Advanced Medicine, Medical Research Institute, Kanazawa Medical University, 1-1 Daigaku, Uchinada, Kahoku 920-0293, Ishikawa, Japan; takajjjj@kanazawa-med.ac.jp (T.T.); takeuchi@kanazawa-med.ac.jp (M.T.)

**Keywords:** advanced glycation end-products (AGEs), glyceraldehyde (GA), glyceraldehyde-derived AGEs, toxic AGEs (TAGE), osteoblasts

## Abstract

In diabetic patients, the metabolism of excess glucose increases the toxicity of the aldehyde group of sugar. Aldehydes, including glyceraldehyde (GA), react with intracellular proteins to form advanced glycation end-products (AGEs), which deteriorate bone quality and cause osteoporosis. One of the causes of osteoporotic fractures is impaired osteoblast osteogenesis; however, the cytotoxic effects of aldehydes and the subsequent formation of AGEs in osteoblasts have not yet been examined in detail. Therefore, the present study investigated the cytotoxicity of intracellular GA and GA-derived AGEs, named toxic AGEs (TAGE), in the mouse osteoblastic cell line MC3T3-E1. Treatment with GA induced MC3T3-E1 cell death, which was accompanied by TAGE modifications in several intracellular proteins. Furthermore, the downregulated expression of Runx2, a transcription factor essential for osteoblast differentiation, and collagen correlated with the accumulation of TAGE. The GA treatment also reduced the normal protein levels of collagen in cells, suggesting that collagen may be modified by TAGE and form an abnormal structure. Collectively, the present results show for the first time that GA and TAGE exert cytotoxic effects in osteoblasts, inhibit osteoblastic differentiation, and decrease the amount of normal collagen. The suppression of GA production and associated accumulation of TAGE has potential as a novel therapeutic target for osteoporosis under hyperglycemic conditions.

## 1. Introduction

Bone is remodeled through continuous resorption by osteoclasts and the replacement of old bone with new bone formed by osteoblasts. The differentiation and maturation of osteoblasts are essential for the formation of new bone, with the depletion of osteoblasts inducing osteoporosis. Diabetes mellitus (DM) has been identified as one of the risk factors for osteoporotic fractures. Previous studies demonstrated that the risk of fractures was increased in patients with type 1 or type 2 DM [1,2]. Fractures significantly reduce quality of life and worsen life expectancy [3]. Therefore, the mechanisms underlying cell death in osteoblasts have been extensively examined. Under hyperglycemic conditions, advanced glycation end-products (AGEs) are generated through a Maillard reaction between the aldehyde group of sugar and amino groups of proteins [4]. The formation of AGEs is irreversible, and their accumulation eventually causes tissue damage. In bone tissue, the accumulation of AGE cross-links has been associated with bone strength loss in diabetic model rats and type 1 DM patients with persistent hyperglycemia [5,6]. The physiological cross-linking of collagen is important for the strength and proper biological function of bone; however, collagen cross-linked by AGEs reduces the physical and mechanical properties of tissue components [7,8]. AGEs exhibit a number of cytotoxic activities and have been shown to damage osteoblasts. Research on AGEs has focused on the following two main pathways: (1) extracellular AGEs exert their effects inside of cells through RAGE, a receptor for AGEs, and (2) proteins in cells are modified by AGEs and become dysfunctional. Osteoblasts express RAGE and, thus, are affected by extracellular AGEs. The expression of RAGE was previously shown to be upregulated on the membranes of osteoblasts cultured with AGEs. Furthermore, extracellular AGEs induced apoptosis in cultured osteoblasts [9]. Extracellular AGEs have also been shown to promote downregulated expression of genes that are important for osteoblast differentiation, such as Runx2 and osterix, and inhibit calcification [10,11].

While extracellular AGEs impair the function of osteoblasts via RAGE, they are present not only extracellularly but also intracellularly. The cellular intake of glucose and fructose increases under hyperglycemic conditions, and metabolism proceeds with the production of several metabolic intermediates [12]. Some of these intermediates are aldehydes, which are electrophilic compounds that induce cytotoxicity [13]. Furthermore, these aldehyde groups, including glyceraldehyde (GA), easily react with proteins and form AGEs. Previous studies demonstrated that the expression of osteoblastic markers was downregulated in the mouse osteoblast-like cell line MC3T3-E1 cultured under high glucose conditions [14,15], suggesting that the metabolism of excess glucose suppressed osteoblast differentiation. In chondrocytes and osteoblasts, intracellular glycolaldehyde-derived AGEs have been reported to induce cell death via endoplasmic reticulum stress [16,17]. In addition to glycolaldehyde, GA, an intermediate of glucose metabolism, forms AGEs. We previously prepared a specific antibody to analyze the toxicity of GA-derived AGEs [18]. The antibody was shown to recognize different epitopes from GA-derived structures, such as 3-hydroxy-5-hydroxymethyl-pyridinium (GLAP) and triosidines [19,20]. The antibody also did not recognize various AGEs generated from reducing sugar/carbonyl molecules [18]. Meanwhile, serum AGEs from diabetic patients were found to exert neurotoxic effects, which were only neutralized by the addition of the anti-TAGE antibody [21]. In other words, unknown AGE structures derived from GA that are recognized by the anti-TAGE antibody may exhibit cytotoxicity. Based on these findings, we refer to the AGEs recognized by the anti-TAGE antibody as toxic AGEs (TAGE). Increased TAGE levels were found to correlate with various lifestyle diseases, such as non-alcoholic steatohepatitis, DM, cancer, dementia, infertility, and cardiovascular diseases [22,23,24]. An analysis of the toxicity of TAGE in the liver, a major site for the metabolism of excess glucose and fructose, revealed that the accumulation of TAGE was associated with damage to cell function and the induction of cell death, ultimately leading to liver disease [25,26,27,28]. Glucose metabolism is also performed by osteoblasts [29]. The production of GA and accumulation of TAGE in osteoblasts are expected to have a negative impact on bone function in patients with DM, and an analysis of these factors in osteoblasts may lead to a more detailed understanding of diabetic osteoporosis.

Therefore, we herein investigated the effects of GA and the associated accumulation on TAGE in MC3T3-E1 cells.

## 2. Materials and Methods

### 2.1. Reagents and Antibodies

GA and aminoguanidine (AG) were purchased from Nacalai Tesque (Kyoto, Japan) and Wako (Osaka, Japan)., respectively. Anti-collagen type 1 α 1 (COL1A1; sc-293182) and anti-β-tubulin (014-25041) antibodies were from Santa Cruz (CA, USA) and Wako, respectively. A horseradish peroxidase (HRP)-conjugated secondary antibody was purchased from Cell Signaling Technology (MA, USA). An anti-TAGE antibody was prepared and purified as previously described [18].

### 2.2. Cell Culture

The mouse osteoblast-like cell line MC3T3-E1 (ECACC No. 99072810) was purchased from ECACC. MC3T3-E1 cells were grown in α-MEM (Sigma, MI, USA) supplemented with 10% fetal bovine serum (FBS; Sigma), 100 U/mL penicillin, 100 µg/mL streptomycin (Wako), and 2 mM L-glutamine (Sigma). MC3T3-E1 cells were plated at a density of 1.5 × 10^4^ cells/cm^2^.

### 2.3. Cell Viability

Cell viability was assessed using the CellTiter-Glo luminescent cell viability assay according to the manufacturer’s instructions (Promega, WI, USA). In opaque 96-well plates, cells were plated in three different wells for each desired reagent treatment and incubated for 24 h. After the incubation, cells were treated with (1, 2, or 4 mM) or without AG for 2 h and then with (1 or 2 mM) or without GA for 24 h. AG was used to inhibit the formation of AGEs by reacting with GA. The viability of the treated cells was measured using the CellTiter-Glo reagent. Experimental values were subtracted from the fluorescence values of the wells containing medium with the drug in the absence of cells as the background. Neither GA nor AG affected luciferase activity in this assay.

### 2.4. Slot Blot Analysis

The total amount of TAGE in MC3T3-E1 cell extracts treated with reagents was measured by a slot blot analysis, which was performed as previously reported [26]. Briefly, cell extracts were prepared using lysis buffer (2 M thiourea, 7 M urea, 30 mM Tris, 4% CHAPS, and protease inhibitor cocktail (complete Mini; Roche, Basel, Switzerland)). PVDF membranes fixed with cell lysates using a slot blot apparatus (Bio-Rad, CA, USA) were incubated with the anti-TAGE antibody (1:1000) and then with HRP-conjugated secondary antibody (1:2000) at room temperature (R.T.) for 1 h. Chemi-Lumi One Super (Nacalai Tesque) was used to detect immunoreactive proteins, and proteins were scanned using Fusion (Vilber Lourmat, Marne La Vallee, France).

### 2.5. Western Blot Analysis

MC3T3-E1 cells were lysed in Laemmli sample buffer (Bio-Rad). Samples were then heated to 95 °C for 5 min. The same concentrations of proteins from cell extracts were separated on SDS-polyacrylamide gels. These proteins were transferred to PVDF membranes, which were then incubated with the anti-TAGE antibody (1:1000) or anti-COL1A1 antibody (1:2000) at 4 °C overnight followed by the secondary antibody (1:2000) at R.T. for 1 h. Chemi-Lumi One Super was used to detect immunoreactive proteins, and protein bands were scanned using Fusion. The anti-β-tubulin antibody at a 1:30,000 dilution was used as an internal reference.

### 2.6. Real-Time Quantitative PCR (qPCR)

The RNeasy Micro Kit (Qiagen, Hilden, Germany) was used to extract RNA. After isolation, 1 µg of total RNA from each sample was reverse transcribed using the ReverTra Ace qPCR RT Master Mix (Toyobo, Osaka, Japan) according to the manufacturer’s guide. The TaqMan Gene Expression Assay (Thermo Fisher Scientific, Waltham, MA, USA) was adopted for the gene expression analysis of Runx2 (Mm00501584_m1), osterix (Mm00504574_m1), alkaline phosphatase (ALP, Mm00475834_m1), and COL1A1 (Mm00801666_g1) in a QuantStudio™ 12K Flex Real-Time PCR System (Thermo Fisher Scientific). Expression levels were assessed using the ∆∆CT method. GAPDH (Mm99999915_g1) was used as an internal standard.

### 2.7. Immunofluorescence Staining

MC3T3-E1 cells grown on 12-millimeter coverslips were treated with (4 mM) or without AG for 2 h followed by treatment with (1 mM) or without GA for 24 h. Cells were treated with 4% paraformaldehyde for 15 min for fixation. These cells were permeabilized with 0.5% Triton X-100/PBS for 5 min. Fixed cells were incubated with the anti-COL1A1 antibody (1:50) at 4 °C overnight, followed by a secondary antibody conjugated to a fluorescent dye (Alexa Fluor 488, Thermo Fisher Scientific) at R.T. for 1 h. Nuclei were visualized with DAPI (4,6-diamidino-2-phenylidole). Cell images were captured using a BZ-X700 microscope (Keyence, Osaka, Japan).

### 2.8. Statistical Analysis

Results are expressed as the mean ± S.D., and experiments were repeated at least three times. Statistical analyses were performed using Stat Flex 6.0 software (Artech, Osaka, Japan). A one-way ANOVA followed by Tukey’s test was used to compare differences among samples. Significant differences are shown as *p*-values < 0.05 and <0.01 in the figures.

## 3. Results

### 3.1. MC3T3-E1 Cell Death Associated with the Accumulation of TAGE

**Figure 1:**
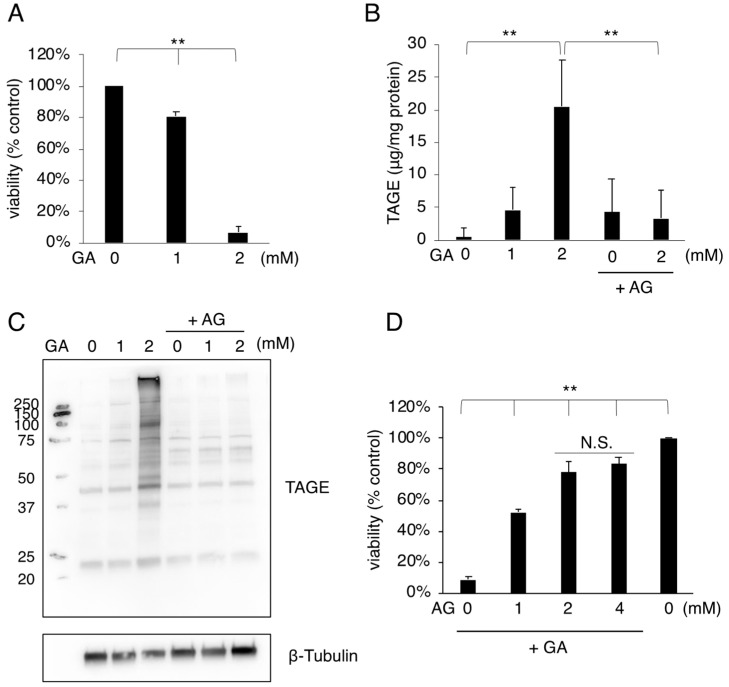
Cell death and TAGE accumulation were induced in MC3T3-E1 cells treated with GA. (**A**) Dose-dependent effects of GA on the viability of MC3T3-E1 cells (*n* = 3). MC3T3-E1 cells were treated with 0, 1, or 2 mM GA for 24 h. (**B**) The total amount of TAGE in MC3T3-E1 cells was measured using an anti-TAGE antibody (*n* ≥ 3). MC3T3-E1 cells were treated with (4 mM) or without AG for 2 h followed by with (1 or 2 mM) or without GA for 24 h, and cell extracts were then prepared. (**C**) Western blot analysis of TAGE in MC3T3-E1 cells. Cell extracts were prepared from MC3T3-E1 cells after a treatment with (4 mM) or without AG for 2 h and then incubated with (1 or 2 mM) or without GA for 24 h. Proteins were probed with an anti-TAGE antibody. β-Tubulin was used as a loading control. Experiments were independently conducted three times, and similar results were obtained (Figure S1). (**D**) Cell death induced by the GA treatment was rescued by the AG pretreatment (*n* = 3). MC3T3-E1 cells were treated with AG (0, 1, 2, or 4 mM) for 2 h and then with (2 mM) or without GA for 24 h. Results are expressed as the mean ± S.D. A one-way ANOVA followed by Tukey’s test was used to compare all samples (** *p* < 0.01 and not significant (N.S.)) (**A**,**B**,**D**). GA; glyceraldehyde, AG; aminoguanidine, TAGE; toxic advanced glycation end-products.

We initially performed a cell viability assay using mouse osteoblastic MC3T3-E1 cells treated with GA because cell death in osteoblasts is one of the most important causes of osteoporosis. The viability of MC3T3-E1 cells decreased in a GA-dose-dependent manner from 1 mM GA (Figure 1A). GA reacts with proteins to produce TAGE, which damage proteins and cell functions. Therefore, we investigated whether TAGE accumulated in MC3T3-E1 cells treated with GA. Cell extracts from GA-treated MC3T3-E1 cells were examined to quantify intracellular TAGE using a slot blot analysis with the anti-TAGE antibody. TAGE accumulation in MC3T3-E1 cells was markedly increased by 2 mM GA (Figure 1B). Furthermore, we examined the composition of TAGE-modified proteins in GA-treated cells using a Western blot analysis with the anti-TAGE antibody. The treatment with 2 mM GA increased the intensities of several TAGE-modified protein bands in MC3T3-E1 cell lysates (Figure 1C). We then used AG in combination with GA. AG has been reported to inhibit the formation of AGEs by reacting with aldehydes [30]. Pretreatment with AG suppressed GA-induced increases in intracellular TAGE (Figure 1B). Moreover, the increased intensities of TAGE bands with the GA treatment were reduced by the pretreatment with AG (Figure 1C). These results suggest that TAGE production was sufficiently inhibited by the AG pretreatment in MC3T3-E1 cells. In addition, cell death induced by the GA treatment was suppressed by the AG pretreatment (Figure 1D), indicating that the accumulation of TAGE correlated with MC3T3-E1 cell death.

### 3.2. Downregulated Expression of Runx2 in GA-Treated MC3T3-E1 Cells

**Figure 2:**
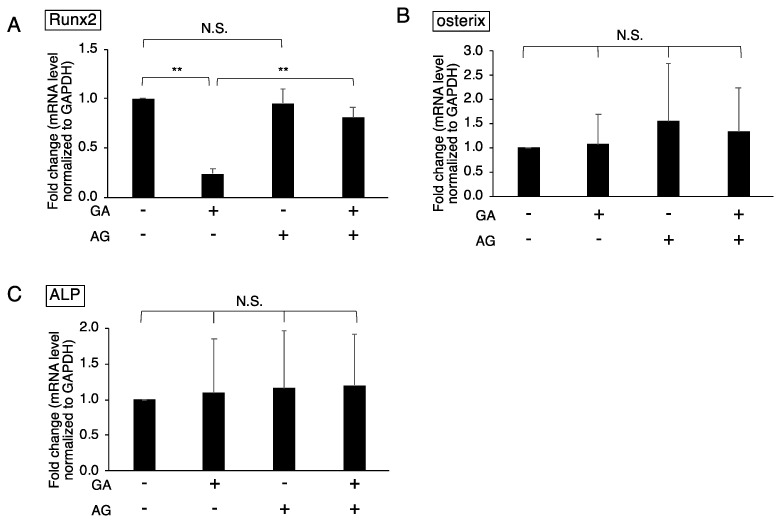
Effects of the GA treatment on the expression of genes involved in osteoblastic differentiation in MC3T3-E1 cells. Cells were treated with (4 mM) or without AG for 2 h followed by with (2 mM) or without GA for 24 h, and total RNA was then extracted. Each experiment was independently performed at least three times in three wells. The expression levels of Runx2 (**A**), osterix (**B**), and ALP (**C**) are shown as a ratio to that of GAPDH. Results are expressed as the mean ± S.D. of fold changes from the no treatment value. A one-way ANOVA followed by Tukey’s test was used to compare all samples (** *p* < 0.01 and not significant (N.S.)). GA; glyceraldehyde, AG; aminoguanidine, ALP; alkaline phosphatase, GAPDH; glyceraldehyde-3-phosphate dehydrogenase.

Osteoblast differentiation is an essential factor in normal bone formation. To clarify whether osteoblast differentiation is inhibited by GA and the subsequent accumulation of TAGE, the expression of osteogenic markers, including Runx2, osterix, and ALP, in GA-treated MC3T3-E1 cells was investigated using real-time qPCR. The expression of Runx2, a transcription factor essential for osteoblast differentiation, was suppressed by the GA treatment for 24 h (Figure 2A). We then examined the effects of AG on Runx2 expression in cells treated with GA. GA-induced decreases in expression levels were attenuated by the AG pretreatment (Figure 2A). In contrast, the expression of osterix, another transcription factor, and ALP, an important protein for calcification, was not affected by the GA treatment (Figure 2B,C). These results suggest that the downregulated expression of Runx2 correlated with the accumulation of TAGE. On the other hand, the regulation of osterix and ALP expression was not affected by GA or the accumulation of TAGE.

### 3.3. Effects of the GA Treatment on COL1A1 in MC3T3-E1 Cells

**Figure 3:**
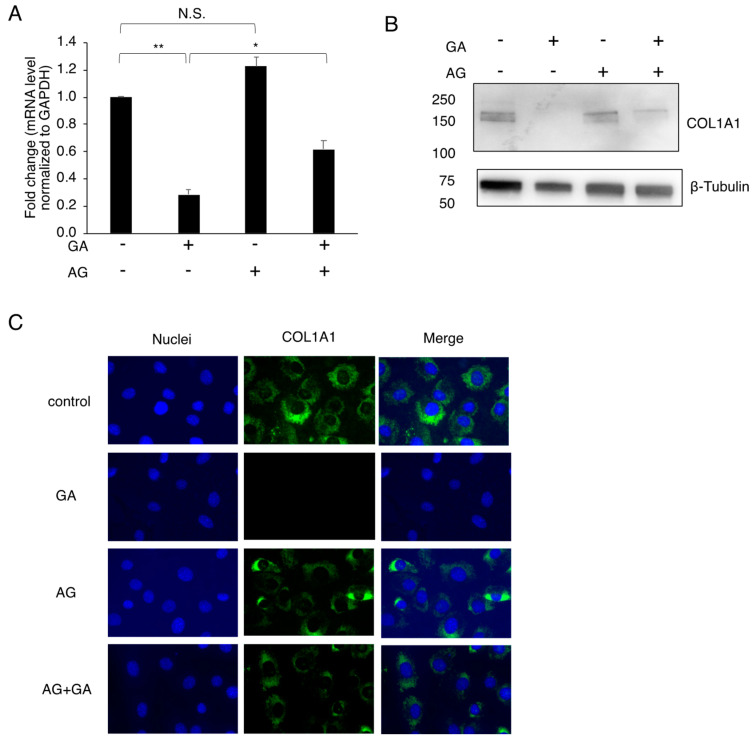
Effects of the GA treatment on the expression and detection of collagen in MC3T3-E1 cells. (**A**) A quantitative RT-PCR analysis of COL1A1 mRNA expression in MC3T3-E1 cells treated with (4 mM) or without AG for 2 h followed by with (2 mM) or without GA for 24 h. Each experiment was independently performed three times in three wells. Results are expressed as the mean ± S.D. fold changes from the no treatment value. A one-way ANOVA followed by Tukey’s test was used to compare all samples (** *p* < 0.01, * *p* < 0.05, and not significant (N.S.)). (**B**) Western blot analysis of the intracellular COL1A1 protein. MC3T3-E1 cells were treated with (4 mM) or without AG for 2 h followed by with (2 mM) or without GA for 24 h, and cell extracts were then prepared. β-Tubulin was used as the loading control. Experiments were independently conducted three times, and similar results were obtained (Figure S2). (**C**) Fluorescence images of nuclei (blue fluorescence) and COL1A1 (green fluorescence) in MC3T3-E1 cells treated with (4 mM) or without AG for 2 h followed by with (1 mM) or without GA for 24 h. The magnification for the figure is ×40. Experiments were repeated twice with similar results (Figure S3). GA; glyceraldehyde, AG; aminoguanidine, COL1A1: collagen type 1α1, GAPDH; glyceraldehyde-3-phosphate dehydrogenase.

Collagen 1, which is abundant in osteoblasts, was previously shown to be modified by AGEs under diabetic conditions [7,8]. Therefore, we investigated the effects of the GA treatment on COL1A1 in MC3T3-E1 cells. We initially examined the expression of COL1A1 in MC3T3-E1 cells and found that its mRNA expression level was decreased by the GA treatment for 24 h (Figure 3A). The reduction was restored by AG, suggesting that the downregulated expression of COL1A1 correlated with the accumulation of TAGE. The behavior of collagen 1 proteins in MC3T3-E1 cells was then examined using a Western blot analysis. The GA treatment rendered the protein band of COL1A1 in untreated cells undetectable by the COL1A1 antibody, and the combined use of AG restored its detection (Figure 3B). A fluorescence immunostaining analysis using the anti-COL1A1 antibody also failed to detect collagen proteins in GA-treated cells (Figure 3C). The inability to detect COL1A1 was restored by the treatment with AG combined with GA in MC3T3-E1 cells. These results suggest that collagen was modified by TAGE in cells treated with GA, causing a structural change that prevented its recognition by the anti-COL1A1 antibody.

## 4. Discussion

Glucose and fructose are metabolized and used as energy; however, some of the metabolic intermediates produced during this process have aldehyde groups. GA, one of the aldehyde groups, is classed as an electrophilic compound that damages cells. GA also binds to intracellular proteins to form GA-derived AGEs, which are called TAGE because of their cytotoxicity. A positive correlation has been reported between the increased accumulation of TAGE and various lifestyle-related diseases [22,23]. TAGE are formed and accumulate in organs that metabolize glucose and fructose. The accumulation of TAGE in organs, including the liver, brain, and pancreas, has been suggested to contribute to the pathogenesis of diseases [22,23]. In addition to these organs, bone metabolizes glucose, which may produce GA, a factor contributing to the accumulation of TAGE. The accumulation of AGEs was previously reported in the bone tissue of spontaneously diabetic rats and type 1 DM patients [5,6]. AGEs induce protein dysfunction due to abnormal cross-linking structures within and between proteins [31]; however, the toxicity of the intermediates of glucose and fructose metabolism and AGEs in osteoblasts has not yet been examined in detail. Furthermore, the toxicities of GA and TAGE remain unknown. We herein investigated the cytotoxicity of GA in MC3T3-E1 cells and found that cell death was associated with the accumulation of TAGE (Figure 1). Intracellular TAGE-modified proteins were examined using a Western blot analysis, and the results obtained showed that several proteins were modified by TAGE (Figure 1C). The target of TAGE-modified proteins will be important for elucidating the pathogenesis of disease, and we intend to investigate TAGE-modified proteins and their dysfunction in the future.

In addition to the death of osteoblasts, the inhibition of osteoblastic differentiation has been shown to contribute to DM-related bone loss. Runx2 is a transcription factor for osteoblastic differentiation [32]. It upregulates the expression of bone matrix protein genes during osteoblast differentiation. The present results revealed that the expression of Runx2 was downregulated by the GA treatment (Figure 2A). The GA-induced downregulation of Runx2 was suppressed by the AG pretreatment, indicating a relationship between the regulation of Runx2 expression and TAGE accumulation. Downregulated expression of Runx2 has been detected in MC3T3-E1 cells grown under high glucose culture conditions [14,15]. Previous studies using animal models of hyperglycemia also reported low expression levels of Runx2 [33]. Under high glucose conditions, GA production and the subsequent formation of TAGE may contribute to the downregulated expression of Runx2. Similar to Runx2, osterix was also shown to be suppressed in high glucose medium and hyperglycemic animal models [14,15,33]. However, no changes were observed in the expression of osterix in MC3T3-E1 cells treated with GA (Figure 2B). ALP is also necessary for bone calcification and is a frequently used marker of the osteoblast differentiation process. Its activity was previously shown to be decreased in MC3T3-E1 cells grown under high glucose culture conditions and in a diabetic state in animal models [14,33]. However, ALP in MC3T3-E1 cells was not affected by the GA treatment, similar to osterix (Figure 2C). Differentiation markers such as Runx2 and osterix exhibit the same behavior in response to high glucose conditions, including hyperglycemia. Yet in the present study, Runx2 expression was repressed in cells treated with GA, whereas other differentiation markers were not affected. Osterix is also a transcription factor that promotes osteoblast differentiation, and its expression is regulated by Runx2 and Runx2-independent pathways [34]. Therefore, the expression of osterix under GA-treated conditions may be independent of Runx2. Since we only investigated the differentiation of osteoblasts in a short time period in the present study, further research is needed to examine it in a longer time period under conditions that promote the accumulation of GA and TAGE. However, the results obtained suggest a new possibility that GA and/or TAGE and other factors work in different pathways to signal osteoblastic differentiation under hyperglycemic conditions.

Besides these markers of differentiation, the downregulated expression of collagen was previously reported under high glucose culture conditions and in hyperglycemic animal models [15,33]. In the present study, the suppression of COL1A1 expression in MC3T3-E1 cells correlated with the accumulation of TAGE (Figure 3A). Furthermore, COL1A1 expression in GA-treated MC3T3-E1 cells was almost undetectable by the anti- COL1A1 antibody (Figure 3B,C). The metabolic rate of collagen is very low, and its turnover in bone takes approximately 1–2 years [35]. Therefore, it is unlikely that collagen was almost completely degraded in GA-treated MC3T3-E1 cells after 24 h in the present study. AGEs have been shown to accumulate in bone tissue and modify collagen [7,8]. In the present study, the AG pretreatment increased the detectability of COL1A1 in cells treated with GA. Therefore, the structure of COL1A1 may have been modified by TAGE and, thus, was no longer recognized by the anti-COL1A1 antibody. Further studies are needed to clarify TAGE-induced modifications in collagen.

The GA treatment led to various dysfunctions in MC3T3-E1 cells; however, it currently remains unclear whether GA or TAGE exert more adverse effects. GA is highly reactive and has been shown to react with proteins in vitro to form TAGE-modified proteins within a few hours [26]. Once proteins have been modified as AGEs, including TAGE, they form abnormal cross-linked structures, and in the case of enzymatic proteins, this inhibits enzymatic activity [26]. AGEs are not easily degraded, and the effects of AGE-modified proteins, particularly long-lived collagen, are expected to be prolonged [36]. In the present study, we only observed the effects of GA and TAGE in MC3T3-E1 cells for a short period of time; however, the long-term effects of GA on several proteins, including collagen, are expected to contribute to the formation of TAGE and diversely affect bone formation.

**Figure 4:**
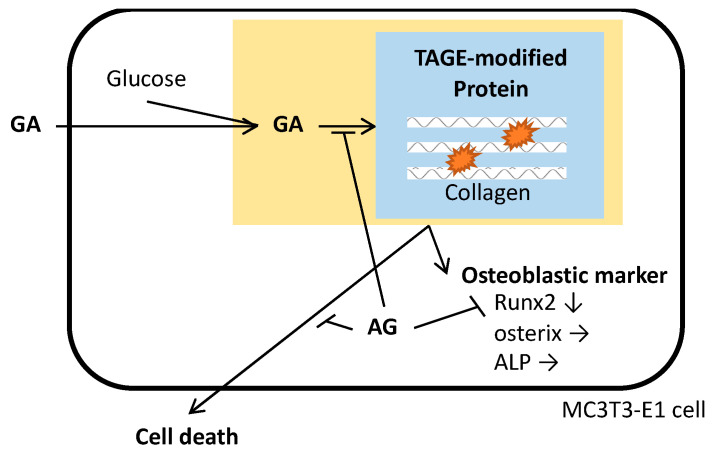
A schematic representation of GA and TAGE effects on MC3T3-E1 cells. GA-induced TAGE modifications in intracellular proteins, including collagen, eventually resulted in cell death. The GA treatment and TAGE accumulation correlated with the down-regulated expression of Runx2. GA; glyceraldehyde, AG; aminoguanidine, TAGE; toxic advanced glycation end-products, ALP; alkaline phosphatase.

The present study is the first to report a relationship between GA and the subsequent accumulation of TAGE and osteoblast cell damage (Figure 4) and provides novel insights into the role of TAGE in the pathogenesis of osteoporotic fractures associated with DM.

## Data Availability

Data is contained within the article or supplementary material.

## Supplementary Materials

The following are available online at https://www.mdpi.com/article/10.3390/nu14050990/s1, Figure S1. Western blot analysis of TAGE in MC3T3-E1 cells. Cell extracts were prepared from MC3T3-E1 cells after a treatment with (4 mM) or without AG for 2 h and then incubated with (1 or 2 mM) or without GA for 24 h; Figure S2. Western blot analysis of the intracellular COL1A1 protein. MC3T3-E1 cells were treated with (4 mM) or without AG for 2 h followed by with (2 mM) or without GA for 24 h, and cell extracts were then prepared; Figure S3. Fluorescence images of nuclei (blue fluorescence) and COL1A1 (green fluorescence) in MC3T3-E1 cells treated with (4 mM) or without AG for 2 h followed by with (1 mM) or without GA for 24 h. The magnification for the figure is ×40.
