# Supplementary material for: The Association between Accumulation of Toxic Advanced Glycation End-Products and Cytotoxic Effect in MC3T3-E1 Cells
Source: Nutrients. 2022 Feb 26;14(5):990. doi: 10.3390/nu14050990 (PMC8912344; doi:10.3390/nu14050990)
Supplement: Supplementary file 1 [file nutrients-14-00990-s001.zip › nutrients-1602680-supplementary material.pdf]

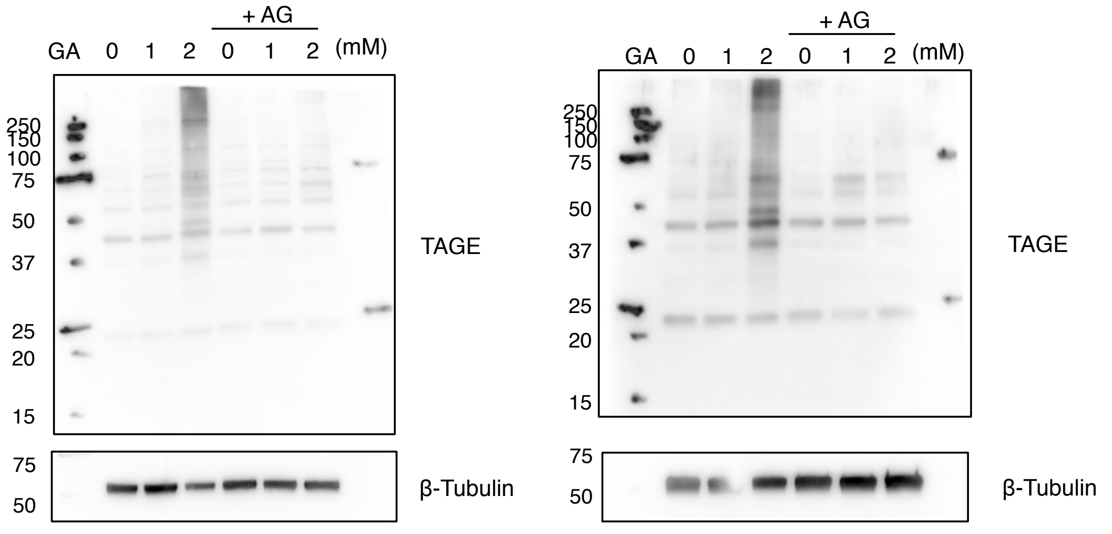

Figure S1. Western blot analysis of TAGE in MC3T3-E1 cells. Cell extracts were prepared from MC3T3-E1 cells after a treatment with (4 mM) or without AG for 2 h and then incubated with (1 or 2 mM) or without GA for 24 h. Proteins were probed with an anti-TAGE antibody. β-Tubulin was used as a loading control.

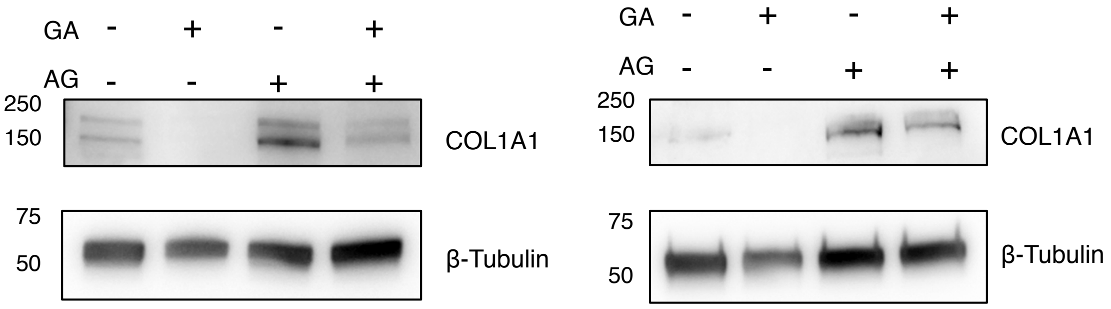

Figure S2. Western blot analysis of the intracellular COL1A1 protein. MC3T3-E1 cells were treated with (4 mM) or without AG for 2 h followed by with (2 mM) or without GA for 24 h, and cell extracts were then prepared. β-Tubulin was used as the loading control

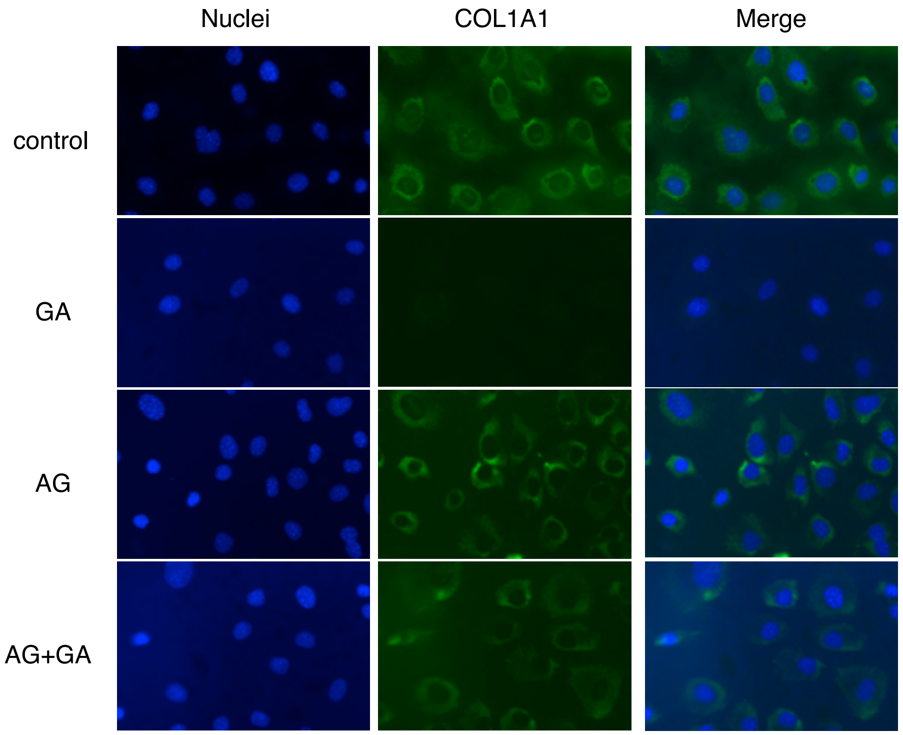

Figure S3. Fluorescence images of nuclei (blue fluorescence) and COL1A1 (green fluorescence) in MC3T3-E1 cells treated with (4 mM) or without AG for 2 h followed by with (1 mM) or without GA for 24 h. The magnification for the figure is ×40.
